# Glycemic response to SSBs and ASBs: the role of mixed meals and individual variability

**DOI:** 10.1186/s12937-025-01181-x

**Published:** 2025-07-16

**Authors:** Sejin Kim, YoonJu Song

**Affiliations:** Department of Food Science & Nutrition, The Catholic University of Korea, 43 Jibong-ro, Wonmi-gu, Bucheon-si, 14662 Geonggi-do Korea, Republic of

**Keywords:** Sugar sweetened beverages, Artificially sweetened beverages, Postprandial glycemic response, Continuous glucose monitoring

## Abstract

**Background:**

While artificially sweetened beverages (ASBs) are widely reported to have minimal glycemic impact compared to sugar-sweetened beverages (SSBs), their effects in mixed meal conditions and individual variability in response remain poorly understood. This study aimed to evaluate postprandial glycemic response (PPGR) and individual variability in response to an SSB (regular cola) and an ASB (zero cola), both in single and mixed conditions, using continuous glucose monitoring (CGM).

**Methods:**

A total of 66 healthy young adults participated in this 14-day, non-randomized crossover intervention study. Test meals included 75 g oral glucose load as a reference, muffin, regular cola, zero cola, muffin with regular cola (MRC), and muffin with zero cola (MZC). PPGR was evaluated using incremental area under the curve. The glucose dip was assessed as the minimum glucose reduction from baseline. Participants were classified as MZC-High (*n* = 17) if their glycemic response to MZC was higher than to MRC, and as MZC-Stable (*n* = 44) if MRC showed the higher response.

**Results:**

The 75 g oral glucose load reference exhibited a typical glycemic pattern, peaking at 45 min before steadily declining. The muffin induced a moderate glycemic response, while regular cola led to a rapid glucose rise followed by a sharp decline. When combined with a muffin, MRC exhibited a slightly higher glycemic response (iAUC_180_:161.6 mmol∙min/L), whereas MZC showed a similar response to the muffin alone (113.3 and 111.1 mmol∙min /L, respectively). At 120 min, the glucose dip was most pronounced for regular cola, whereas oral glucose load and muffin showed smaller reductions. These patterns persisted at 180 min, with oral glucose load showing the largest drop. Mixed meals attenuated glucose dips, with MRC and MZC preventing excessive declines. Individual responses analysis revealed that while the overall iAUC was not significantly different between muffin alone and MZC, 26 participants (MZC-High Responders) exhibited a higher iAUC with MZC than with MRC, suggesting variability in glucose regulation. Comparisons between MZC-High Responders and MZC-Stable participants showed no significant differences in age or body composition.

**Conclusion:**

While zero cola alone or in combination with a muffin had a minimal overall glycemic impact, some individuals exhibited higher glycemic responses in mixed conditions. These findings suggest that individual variability and mixed condition should be considered when consuming artificially sweetened beverages.

**Trial registration:**

Clinical Research Information Service (CRIS, cris.nih.go.kr) No. KCT0009921.

**Supplementary Information:**

The online version contains supplementary material available at 10.1186/s12937-025-01181-x.

## Introduction

Higher consumption of SSBs has been consistently associated with an increased risk of cardiometabolic diseases, including metabolic syndrome, type 2 diabetes, and cardiovascular disease (CVD) [1–3]. Additionally, several cohort studies have reported a positive association between SSB consumption and an increased risk of mortality in the general population [4–6]. Meta-analyses further support a dose-response relationship, indicating that each additional serving of SSBs is associated with a proportionally higher risk of cardiometabolic diseases and mortality [7, 8]. Given these consistent adverse effects, ASBs have emerged as a potential alternative to SSBs, with the assumption that they offer a way to reduce sugar intake and improve metabolic outcomes without adverse glycemic effects.

Several intervention studies have specifically examined the glycemic effects of beverages containing artificial sweeteners (AS), including aspartame and acesulfame-K, as well as naturally derived sweeteners such as monk fruit and stevia [9–12]. These studies have generally reported minimal or negligible effects on glycemic regulation, suggesting that ASBs may not have a significant impact on postprandial glucose levels. Furthermore, meta-analyses of randomized clinical trials with intervention periods of at least two weeks [13], or six months [14] indicate that replacing SSBs with ASBs results in modest improvements in body weight and cardiometabolic risk factors without evidence of harm in children and adults.

However, most studies have evaluated ASBs as a direct replacement of a single SSB, whereas in real-world setting, ASBs are typically consumed alongside other foods, where additional factors may influence postprandial glycemic response. Some studies have reported that the response to artificial sweeteners, such as sucralose, combined with carbohydrate consumption impairs insulin sensitivity [15] and differ by obesity status and sex [16]. Additionally, replacing SSBs with ASBs did not significantly alter sweet taste preference compared to replacing them with unsweetened beverages, such as water [17], suggesting that individual variability may influence metabolic and sensory responses to ASBs.

Individual differences in postprandial glycemic responses to identical foods have also been well-documented. A study by Zeevi et al. [18], demonstrated high interpersonal variability in glycemic response to the same test meals, despite strong agreement in repeated measures within individuals. Similarly, another study found that multiple factors, including meal composition, gut microbiota, and lifestyle, contribute to a postprandial response, with meal composition being stronger predictor than genetic factors [19]. These findings emphasize the importance of considering individual variability when evaluating the metabolic effects of ASBs in real-world situations.

This study aimed to compare the glycemic effects of SSBs and ASBs in both single and mixed meal conditions using CGM. By evaluating these beverages in real-world dietary contexts, this study investigates how ASBs influence postprandial glycemic responses and provide evidence-based guidance for their incorporation into daily diets.

## Methods

### Study design and subjects

This non-randomized crossover intervention study was designed to evaluate postprandial glycemic responses to multiple test meals, consumed in a preference-based sequence over a two-week period using CGM. The study included test meals incorporating a sugar-sweetened beverage (regular cola), an artificially sweetened beverage (zero cola), and their combination with a muffin to assess their effects under single and mixed meal conditions.

A CGM device was fitted to each participant for 14 days, during which test meals were administered on separate days following an 8-hour overnight fast. Participants were asked to record a daily log, including meal contents, timing, and sleep duration via an online app. The study design and timeline are illustrated in Supplementary Fig. 1.

Participants were healthy adults aged 18 years or older who met the following inclusion criteria: the ability to wear a CGM device for two weeks, consume the provided test meals, and complete monitoring and daily reporting. Exclusion criteria included individuals who had experienced weight fluctuations greater than 10% in the previous three months. Individuals with skin inflammation or other dermatological conditions that could interfere with CGM sensor placement were also excluded. Additionally, those with food allergies that could prevent participation in dietary intervention were not eligible. Participants who were scheduled for medical examinations involving strong magnetic or electromagnetic radiation during the study period were also excluded. Furthermore, individuals who had been diagnosed with metabolic diseases or were taking medications that could affect glucose metabolism were not included in this study.

This study was approved by the Institutional Review Board of the Catholic University of Korea (1040395-202408-08) and registered with the Clinical Research Information Service (CRIS) (Trial registration no. KCT0009921) on November 11, 2024, in Korea, which is affiliated with the WHO International Clinical Trials Registry Platform (ICTRP).

### Test meals

Test Meals included regular cola (RC) as SSB, zero cola (ZC) as the ASB, and their combination with a muffin. The five test meal conditions were: regular cola alone, zero cola alone, muffin alone, regular cola with a muffin (MRC), and zero cola with a muffin (MZC). In addition to the test meals, each participant underwent an oral glucose tolerance test (OGTT) with 75 g of glucose on day 3 following CGM placement to allow sensor stabilization. Participants were required to visit the study unit on five separate days to consume the assigned test meals following an 8-hour overnight fast. The scheduling was arranged based on participants’ availability, and the meal was prepared according to individual preferences. Participants were also instructed to refrain from consuming any food or beverage other than water during the 180-minute postprandial monitoring period.

The nutritional composition of the test meals is presented in Supplementary Table 1. Carbohydrate contents varied across test meals. Zero cola is a commercially available product, and its sweeteners consist of a blend of sucralose and acesulfame-K with no specific amounts.

### CGM profiles and postprandial glycemic response

A CGM device (Abbott FreeStyle Libre 2; Abbott Diabetes Care, Alameda, CA, USA) was placed on each participant for 14 days to assess glycemic variability and overall glucose trends. The CGM metrics included active time (%), indicating the percentage of time the sensor was functioning during the monitoring period (14 days for the FreeStyle Libre 2 device); mean glucose (mg/dL), calculated over the active monitoring period; glucose management indicator (GMI, %), estimating the equivalent A1C level based on the average CGM glucose levels; and coefficient of variation (CV, %) representing glycemic variability [20].

To evaluate the postprandial glycemic response (PPGR) to test meals, the area under the curve (AUC) was calculated using 13 glucose measurements taken at 15-minutes intervals after the meal consumption. Incremental AUC (iAUC) was defined as the area above baseline glucose, with the baseline set to zero for areas below it. The iAUC was calculated iAUC at 120 min and 180 min using the trapezoidal rule, following the method described by Brous et al. [21].

PPGR data up to 180 min were used to define peak glucose, lowest glucose, and glucose dip. Peak glucose was defined as the maximum glucose value observed between baseline and 180 min post-meal, while lowest glucose was the minimum glucose value observed within the same period. Glucose dip was defined as absolute difference between the baseline glucose value and the minimum observed postprandial glucose value during the 180-minute period, as applied in a previous study [22]. and was expressed as a percentage of the baseline value.

For validation of CGM glucose levels, self-monitored blood glucose (SMBG) measurements were obtained at each test meal administration using a glucometer (CareSens, i-SENS, Inc., Seoul, Korea). To minimize potential errors, participants were rescheduled to return on a different day if the difference between the two values exceeded 10 mg/dL. The Clarke error grid analysis demonstrated 96.2% agreement and is presented in Supplementary Fig. 3.

### Other variables

The basic demographic information, including age and sex was collected at baseline using a questionnaire. Body composition was assessed using bioelectric impedance analysis (InBody 370, InBody Co., Seoul, Korea), from which muscle mass, fat mass, and body fat percentage were obtained. Height and weight were measured, and BMI was calculated as weight (kg) divided by height (m^2^). Waist circumference (WC) was measured using a tape measure at the level of the umbilicus, following standard guidelines.

Daily dietary records were collected through participants’ daily logs over the 14-day study period. Nutrient intake was calculated using a web-based dietary evaluation system (CAN-Pro, The Korean Nutrition Society, Korea). The average intake over the study period was calculated excluding the first and last days, as they did not represent full days. This was used to reflect participants’ usual dietary intake.

### Statistical analysis

All statistical analyses were performed using SAS version (10.1). Continuous variables are presented means ± standard deviations (SD), while categorical variables are reported as frequencies and percentages. Comparison between men and women for the basic demographic and general characteristics were conducted independent t-test for continuous variables. Time out of range was also examined but not included in the analysis due to its low frequency in this healthy population.

To assess differences across test meals in all parameters of postprandial glycemic response, including incremental area under the curve at 120 and 180 min and glucose dip, a linear mixed-model was applied to the repeated-measures within individuals. Tukey-Kramer adjusted pairwise comparisons were subsequently performed to evaluate between meal differences. A linear mixed model analysis was used to assess the effect of meal order, testing day on postprandial glycemic response, alongside the main effect of meal type. The model included fixed effects for meal type, meal order, and testing day plus their interactions, with participant ID as a random effect.

Participants were classified as MZC-High if their glycemic response (iAUC at 180 min) to MZC was higher than to MRC, and as MZC-Stable if MRC elicited the higher response, as typically expected. However, to account for potential CGM measurement errors in classifying participants, participants with small differences between MZC and MRC response were excluded through a censoring approach. After applying this threshold, the MZC-High group (*n* = 17) had a minimum iAUC difference of -16.9 mmol/L, while the MZC-Stable group (*n* = 44) started from a difference of 10.5 mmol/dL. Among all postprandial parameters assessed, iAUC at 180 min was selected because it captures the full postprandial glycemic profile, including glucose dip, especially relevant for liquid test meals like beverages [22]. Between-group differences in demographic characteristics and other continuous variables were analyzed using generalized linear model (GLM), adjusting for age and sex.

## Results

The descriptive characteristics of study participants are presented in Table 1. The average age was 22 years, with 31 men among 66. The average BMI was 23.2 kg/m^2^, but significantly differed by sex (24.8 in males and 21.7 in females). Men also had significantly higher muscle mass and lower body fat percentage compared to women. For 14-day CGM metrics, the mean glucose level was significantly higher in men (102.8 mg/dL) than in women (96.8 mg/dL), while glucose variability (CV) showed no significant difference between sexes. Regarding dietary intake, the average daily energy intake was 1727.5 kcal, with men consuming significantly more than in women (1874.8 kcal v 1597.1 kcal). However, the macronutrient distribution did not differ by sex, with an overall average 50.9% carbohydrate, 16.0% protein, and 33.0% fat.

**Table 1:** The general characteristics and nutrient intake in young adults

	Total (*n* = 66)	Male (*n* = 31)	Female (*n* = 35)	*P* value^1^
Age (years)	22.0 ± 1.9	22.5 ± 2.3	21.5 ± 1.5	0.0471
BMI (kg/m^2^)	23.2 ± 4.2	24.8 ± 4.4	21.7 ± 3.6	0.0024
Muscle mass (kg)	26.3 ± 7.0	32.9 ± 4.2	20.5 ± 2.0	< 0.0001
Fat mass (kg)	18.0 ± 8.5	17.7 ± 10.2	18.3 ± 6.7	0.7975
Body fat percent (%)	27.1 ± 8.9	22.1 ± 8.4	31.5 ± 6.8	< 0.0001
Waist circumference (cm)	75.5 ± 11.8	83.0 ± 11.2	68.8 ± 7.6	< 0.0001
**14-Day CGM profiles**				
Active time (%)	95.8 ± 7.9	94.5 ± 10.9	97.0 ± 3.5	0.2192
Mean glucose (mg/dL)	99.7 ± 7.0	102.8 ± 6.0	96.8 ± 6.6	0.0003
GMI (%)	5.7 ± 0.2	5.8 ± 0.1	5.6 ± 0.2	0.0005
CV (%)	18.3 ± 3.2	17.7 ± 3.0	18.8 ± 3.3	0.1572
**Mean 14-Day Nutrient intake**				
Energy (kcal)	1765.0 ± 311.2	1906.6 ± 299.7	1639.7 ± 267.4	0.0008
Carbohydrate (g)	217.6 ± 42.7	229.3 ± 43.6	207.2 ± 39.7	0.0595
Protein (g)	68.7 ± 15.6	75.5 ± 17.7	62.7 ± 10.5	0.0015
Fat (g)	62.9 ± 12.5	67.2 ± 13.4	59.1 ± 10.5	0.0171
Fiber (g)	13.4 ± 4.0	13.3 ± 3.2	13.5 ± 4.6	0.8853
Sugar (g)	49.2 ± 14.2	48.1 ± 15.3	50.1 ± 13.3	0.4929
Energy from				
Carbohydrate (%)	50.9 ± 4.4	50.4 ± 5.0	51.3 ± 3.9	0.4405
Protein (%)	16.0 ± 2.0	16.5 ± 2.4	15.6 ± 1.6	0.0953
Fat (%)	33.1 ± 3.3	33.1 ± 3.4	33.1 ± 3.2	0.9872

Active time, percentage of time the CGM sensor was functioning during the monitoring period; Mean glucose, average glucose level over the entire active monitoring period; GMI, glucose management indicator; CV, coefficient of variation

^1^All *p*-values were calculated using a t-test

### Postprandial glycemic response

**Fig. 1:**
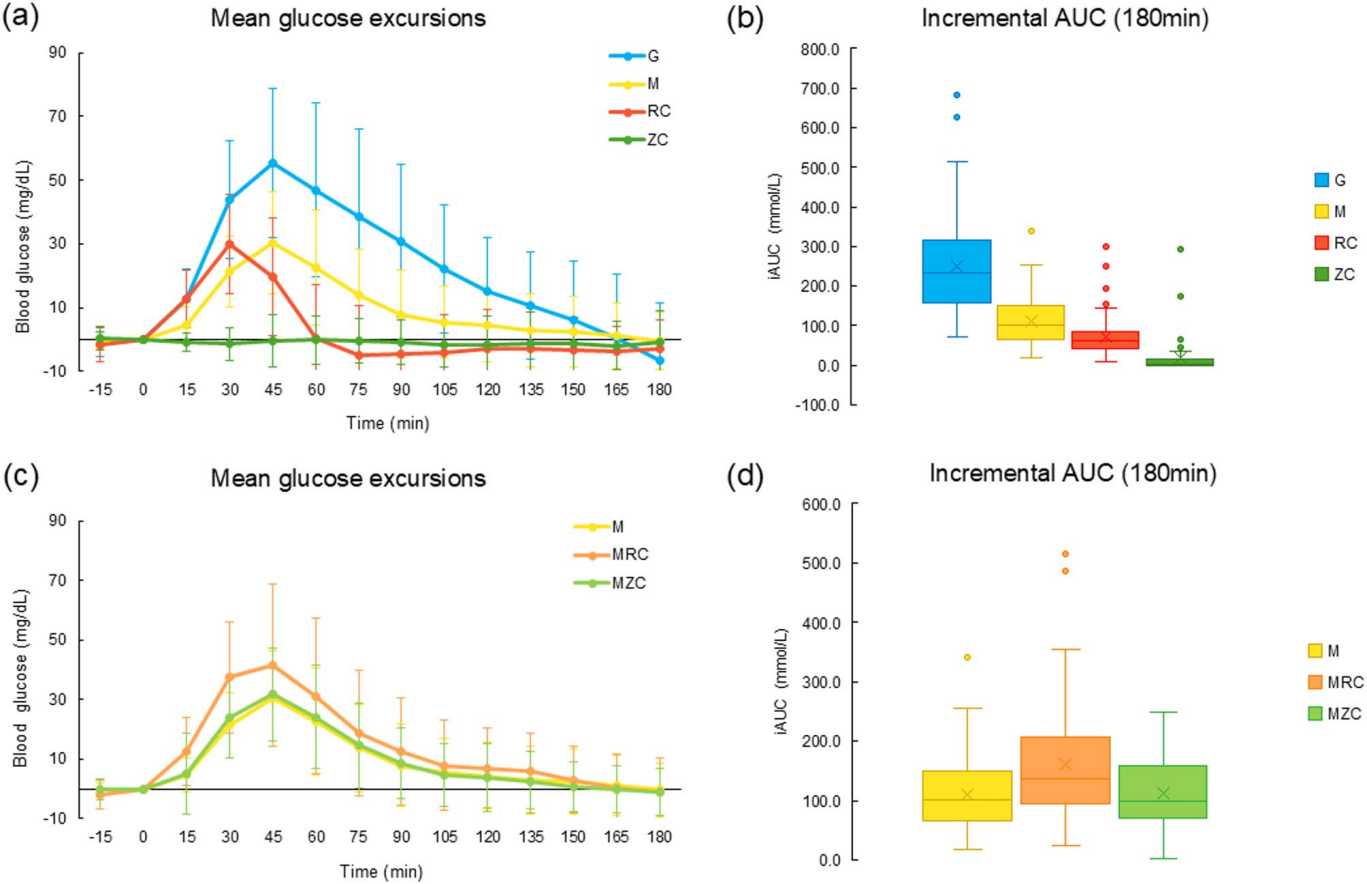
Postprandial glycemic responses to regular and zero cola in single and mixed meal conditions. Mean glucose excursions for (**a**) single meal and (**c**) mixed meal conditions by meal type. Mean incremental AUC values for (**b**) single meal and (**d**) mixed meal conditions by meal type

Figure 1 represents the postprandial glycemic response to test meals, including regular and zero cola, in both single and mixed meal conditions.

The 75 g oral glucose load reference exhibited a typical glycemic pattern, with a gradual rise, peaking at 45 min, followed by a steady decline up to 180 min. In contrast, the muffin (31 g carbohydrates) elicited a moderate glycemic response compared to the 75 g oral glucose load, with the magnitude of response approximately half over the 180-minute period. The regular cola (23 g carbohydrates) induced a rapid glucose increase, peaking at 20 min, followed by a return to baseline at 60 min and a drop below baseline after 75 min. The 3-hour incremental AUC values were 251.2 mmol/L for glucose 75 g, 111.1 mmol/L for the muffin, 72.8 mmol/L for regular cola, and 17.6 mmol/L for zero cola. Mean incremental AUC values differed significantly across meal types (*P* < 0.0001).

When combined with a muffin, muffin with regular cola showed a slightly higher glycemic response than the muffin alone, suggesting a more sustained glucose elevation without rapid glucose dropping. In contrast, muffin with zero cola exhibited a similar glycemic response to the muffin alone. The 3-hour iAUC values were 161.6 mmol/L for muffin with regular cola, 113.3 mmol/L for muffin with zero cola. Mean incremental AUC values differed significantly across meal types, except between muffin and muffin with zero cola, based on Tukey-Kramer adjusted pairwise comparisons from a linear mixed model (*p* < 0.05). Under mixed meal conditions, mean incremental AUC was higher for muffin with regular cola compared to both the muffin alone and the muffin with zero cola (*p* < 0.05).

### Mixed meal effects on glycemic response

**Fig. 2:**
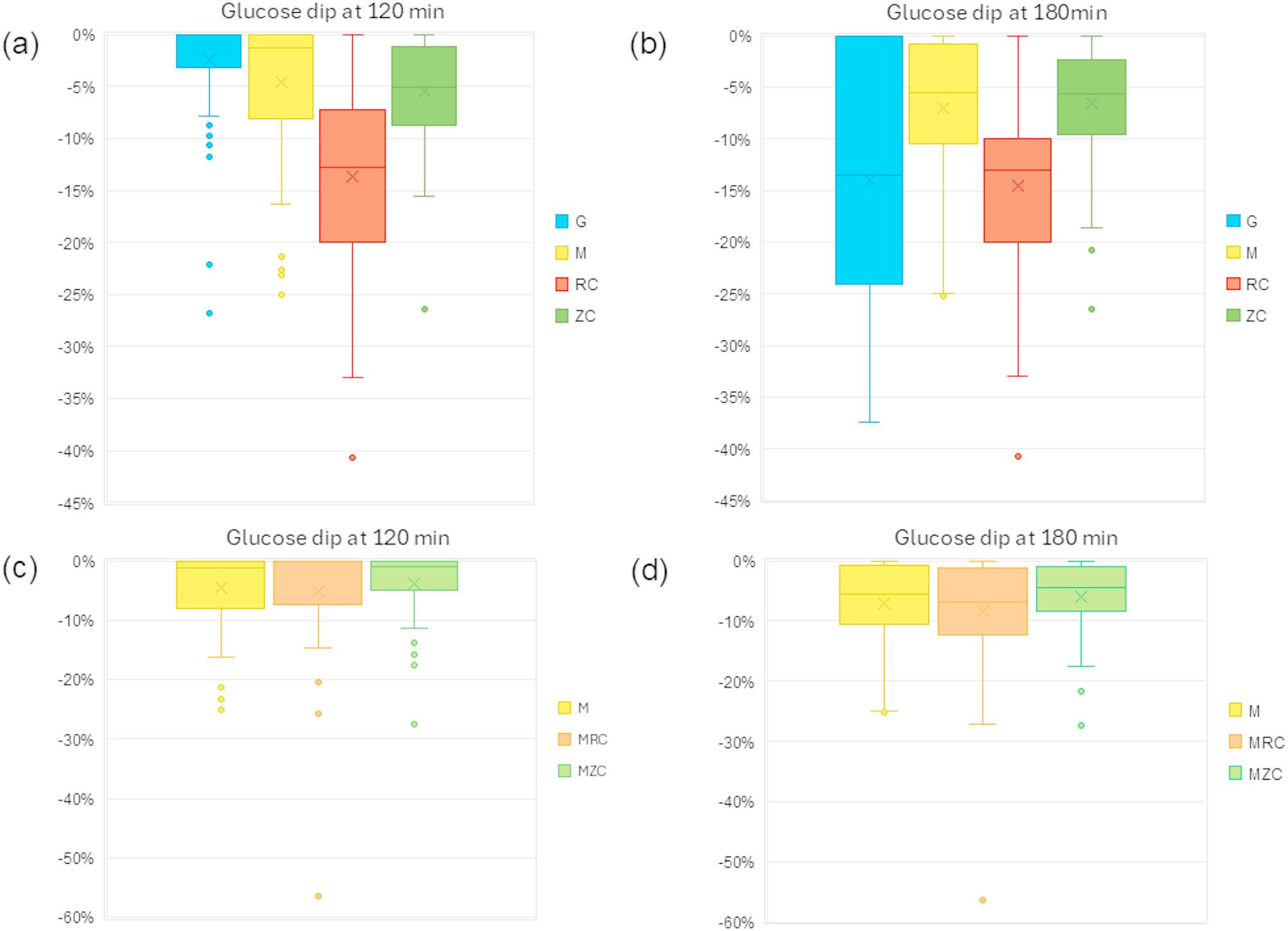
Glucose dip of regular and zero cola in single and mixed meal conditions. Glucose dip was defined as the reduction from baseline glucose levels at (**a**) 120 min and (**b**) 180 min in single meal conditions, and at (**c**) 120 min and (**d**) 180 min in mixed meal conditions

Figure 2 represents the glucose dip, expressed as a percentage of the baseline glucose level, for test meals, including regular and zero cola, in both single and mixed meal conditions.

At 120 min, the average glucose dip from baseline was − 2.4% for oral glucose load, -4.6% for muffin, -13.7% for regular cola, and − 5.5% for zero cola. These declines were largely sustained at 180 min, except for oral glucose load, which showed the largest drop of -14%. The mean percentage of glucose dip differed across meal types. Regular cola and oral glucose load showed significantly greater dips compared to the other meal types (*p* < 0.0001, Tukey-Kramer adjusted). No significant differences in dig magnitude were observed among the remaining meal types.

When consumed with a muffin, the average glucose dip was − 5.1% for muffin with regular cola, -3.8% for muffin with zero cola. These reductions persisted at 180 min, with dips of -8.4% and − 6.0%, respectively, preventing excessive postprandial dip. No significant difference was observed between mixed meal conditions.

To assess potential confounding effects, a linear mixed model was used to evaluate the influence of meal order and testing day on postprandial glycemic response. The analysis showed a significant main effect for meal type (*p* < 0.0001), while meal order (*p* = 0.1001) and testing day (*p* = 0.7091) were not statistically significant in the Supplementary Table 4.

### Individual variability in ASB response

**Fig. 3:**
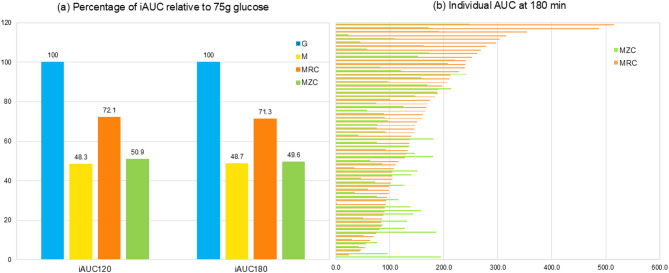
Incremental area under the curve (iAUC) for regular and zero cola in mixed meals. (**a**) Percentage of iAUC for muffin, muffin with regular cola, and muffin with zero cola relative to 75 g oral glucose. (**b**) Individual iAUC values for muffin with regular cola and muffin with zero cola at 180 min. AUC values differed by meal type but not between M and MZC by paired t-test

To evaluate interindividual variability, the percentage of iAUC was calculated relative to each participant’s iAUC for 75 g of glucose, which was designated as 100% (Fig. 3a). On average, the iAUC at 120 min was 48.3% for muffin alone, 72.1% for muffin with regular cola, and 50.9% for muffin with zero cola. At 180 min, the corresponding values were 48.7%, 71.3%, and 49.6%, respectively. Paired comparisons showed no significant differences in iAUC between muffin alone and muffin with zero cola at 120 min (*P* = 0.4129) or 180 min (*P* = 0.7832), indicating that the addition of zero cola did not significantly alter the glycemic response compared to muffin consumption alone.

However, when examining individual responses in mixed meal conditions of muffin with regular or zero cola, variations in glycemic response to muffin with regular cola or zero cola were observed (Fig. 3b). Among these, 21 participants exhibited a higher iAUC for muffin with zero cola than for muffin with regular cola, suggesting variability in postprandial glucose regulation across individuals.

When participants were divided into MZC-High (*n* = 17) and MZC-Stable (*n* = 44) groups, the average age was 21.6 vs. 22.0 years, respectively. The proportion of male participant differed significantly (23.5% vs. 54.6%), based on the chi-square test (Table 2). Body composition measures, including BMI, fat mass, and waist circumference, showed no significant differences between the two groups, although muscle mass showed a marginal difference (*p* = 0.0571). Peak glucose levels following each meal type were not significantly different by the two groups; however, lowest glucose values were significantly lower in the MZC-High responders. In contrast, the 14-Day CGM profiles showed higher mean glucose levels and glucose management indicator in the MZC-Stable groups (*p* < 0.001).

**Table 2:** Baseline characteristics, glycemic response, 14-day CGM data in MZC-Stable and MZC-High responder groups

	MZC-Stable (*n* = 44)	MZC-High responders (*n* = 17)	*P*-value
**Basic and body composition**			
Male (n, %)	24 (54.6%)	4 (23.5%)	0.0293
Age (years)	22.0 ± 1.8	21.6 ± 1.8	0.3840
BMI (kg/m^2^)	23.6 ± 4.6	22.4 ± 3.8	0.3140
Muscle mass (kg)	27.4 ± 7.0	23.6 ± 6.6	0.0571
Fat mass (kg)	18.1 ± 9.1	18.2 ± 8.1	0.9792
Waist circumference (cm)	77.2 ± 12.1	72.0 ± 11.6	0.1317
**Incremental AUC (mmol/L∙180 min)**			
75 g oral glucose load	252.5 ± 122.7	234.2 ± 113.1	0.8432
Muffin	113.2 ± 63.2	112.0 ± 55.3	0.2083
Regular Cola	70.1 ± 44.9	75.1 ± 64.8	0.3720
Zero Cola	14.1 ± 30.4	30.5 ± 70.6	0.6565
Muffin with RC	187.8 ± 101.5	105.1 ± 45.1	0.0126
Muffin with ZC	97.1 ± 57.5	150.2 ± 41.3	0.0057
**Peak glucose (mg/dL)**			
75 g oral glucose load	157.5 ± 24.3	150.4 ± 28.6	0.2494
Muffin	127.5 ± 17.9	118.7 ± 18.5	0.3610
Regular Cola	129.8 ± 16.1	127.0 ± 12.5	0.6818
Zero Cola	96.5 ± 9.9	94.5 ± 11.1	0.1179
Muffin with RC	148.3 ± 22.1	135.9 ± 21.7	0.2649
Muffin with ZC	128.2 ± 15.9	134.4 ± 14.9	0.5915
**Lowest glucose (mg/dL)**			
75 g oral glucose load	80.4 ± 13.1	79.5 ± 12.9	0.4619
Muffin	84.8 ± 8.8	79.9 ± 7.1	0.0689
Regular Cola	80.5 ± 8.6	76.2 ± 9.5	0.0113
Zero Cola	85.3 ± 8.5	82.4 ± 6.3	0.0213
Muffin with RC	85.9 ± 10.1	83.6 ± 12.6	0.7311
Muffin with ZC	90.3 ± 8.5	87.1 ± 8.6	0.2095
**14-Day CGM profiles**			
Active time (%)	96.9 ± 3.7	96.4 ± 4.8	0.6414
Mean glucose (mg/dL)	100.8 ± 6.1	96.9 ± 8.1	0.0009
GMI (%)	5.7 ± 0.1	5.6 ± 0.2	0.0012
CV (%)	18.2 ± 3.3	18.1 ± 2.6	0.6630

MZC, Muffin with Zero Cola; GMI, glucose management indicator; CV, coefficient of variation

^1^ All *p*-values were calculated using a generalized linear model to compare differences between groups, adjusted for age and sex, except for basic and body composition variables

## Discussion

This study examined the glycemic effects of SSBs (regular cola) and ASBs (zero cola) under single and mixed meal conditions using CGM in healthy individuals. The findings demonstrate that SSBs induced the largest glucose dip, but this effect was mitigated in mixed meals, suggesting a stabilizing effect beyond a simple additive response. In contrast, ASBs did not elicit a significant glycemic response in most participants, though individual variability was observed in mixed meal conditions.

### Mixed meal effects on glycemic response to SSBs and ASBs

Few studies have compared postprandial glycemic response to SSBs and ASBs under both single and mixed meal conditions. Glucose dip has been identified as a key factor linking SSB consumption to dysregulated appetite control, increased energy intake, and metabolic disturbance [22–24]. In this study, regular cola induced a rapid glucose rise followed by a sharp decline, exhibiting the highest glucose dip in single meal conditions at 120 min, which persisted at 180 min. Interestingly, while oral glucose load initially produced a smaller glucose dip, by 180 min, it had reached a level comparable to that of regular cola, reinforcing the importance of liquid carbohydrate intake in postprandial glycemic instability.

Excessive SSBs consumption has been associated with an increased risk of metabolic disease, including obesity and cardiovascular disease, as well as higher all-cause and CVD-specific mortality [7, 8]. This heightened risk is partly attributed to the rapid postprandial glucose rise followed by subsequent dip, induced by liquid forms of simple sugars [25, 26]. Glucose dips can be influenced by a variety of physiological mechanisms, including gastric emptying [27], insulin sensitivity [28], and the composition of the gut microbiota [29].

However, this glucose dip phenomenon was mitigated in mixed meal conditions. In this study, the average glucose dips in mixed meal conditions ranged from − 6 to -8% and did not differ by meal type. These values were consistent with the − 6% glucose dip reported for UK average meals [22].

Additionally, the iAUC for muffin with regular cola was approximately 10% lower than the sum of the iAUC for muffin and regular cola consumed separately, indicating that the glycemic response in mixed meal conditions cannot be explained by a simple additive effect. This suggests that food components help buffer the glycemic fluctuations induced by SSBs. When regular cola was consumed with a muffin, the glucose dip was comparable to that of the muffin alone, further supporting the stabilizing effect of mixed meals.

Gastric emptying likely contributes to this buffering effects, as kinetics of glucose appearance differ markedly between solid and liquid foods [30]. Variability in gastric emptying can influence both the timing and magnitude of postprandial glucose excursions, through its effect on nutrient delivery to the small intestine and subsequent insulin release. These findings underscore the importance of evaluating nutritional effects in the context of meals rather than isolated food items.

A few studies have reported that beverages containing artificial sweeteners have minimal effect on postprandial glycemic response over 120 min [11], 24-h glucose profiles [31] and glycemic metabolism over periods of 2 weeks [10] and 12 weeks [12]. Consistent with these findings, zero cola did not elicit a significant glycemic response in either single or mixed meal conditions. When consumed with a muffin, iAUC was not significantly different from that of the muffin alone, and no significant glucose dips were observed. These findings suggest that ASBs may be a viable option for glycemic management, particularly for adults with type 2 diabetes.

### Individual variability in ASB response

However, individual variability in response to ASBs must be considered, particularly in mixed meal conditions. While most participants showed minimal glycemic response to zero cola, but some individuals exhibited a higher iAUC with muffin + zero cola (MZC) than with muffin + regular cola, suggesting unexpected metabolic responses in some individuals. Interestingly, the MZC-High group did not differ significantly in age, BMI, or iAUC of single meal types, although sex distribution differed. The MZC-High groups also exhibited significantly lower nadir glucose following regular cola and zero cola, yet paradoxically slightly lower mean glucose levels and GMI in the 14-day CGM profile. While the mechanism underlying these findings remain unclear, the results underscore the importance of evaluating the effects of ASBs not only within the context of mixed meals, but also through an individualized approach in future studies.

Recent studies suggest that artificial sweeteners may influence glucose tolerance through mechanisms beyond direct glycemic response, including alterations in gut microbiota composition and taste receptor signaling. Emerging evidence indicates that certain sweeteners can induce dysbiosis in the gut microbiome [32], leading to individual-specific, microbiome-dependent changes in glycemic response reported [33]. Additionally, research has shown that sweet taste receptors, expressed in both taste buds and intestinal cells, play a role in glucose absorption and regulation [34, 35]. These findings support growing concern that non-nutritive sweeteners may not be metabolically neutral [36].

Furthermore, the interplay of these multifactorial mechanisms likely contributes to the observed glycemic variability in response to ASBs in this study. Neurological factors, including the reward system, appetite regulation, and sensory perception, also play a role in glycemic homeostasis and postprandial response [15, 16, 31]. These mechanisms may interact differently across individuals, depending on physiological conditions such as insulin sensitivity and gastric emptying.

These findings challenge the widespread assumption that zero cola is an unconditionally suitable alternative to regular cola, particularly in mixed-meal contexts, as some individuals may exhibit unexpected glycemic responses. This highlights the need for an individualized, meal-based approach to dietary guidelines, as neither single food items nor standardized recommendations on ASB consumption fully account for these variations in glycemic response.

### Strengths and limitations

A major strength of this study is the use of continuous glucose monitoring (CGM), which allowed for a detailed analysis of postprandial glycemic responses over an extended period. While CGM has limitations in accuracy and a time lag compared to capillary or venous glucose measurements [37, 38], it offers practical advantages in free-living settings by reducing participant burden and improving adherence. Additionally, the study was designed to compare the glycemic response in both single and mixed meals conditions, which better reflects a real-world dietary setting and provides valuable insights into the interactive effects of ASBs when consumed with food on glycemic regulation.

However, this study has some limitations. First, it was conducted in 66 healthy young adults, which may limit the generalizability of the findings to the broader adult population, particularly to individuals with impaired glycemic regulation. Second, all glucose values were obtained using CGM, with each test meal assessed once, which may limit accuracy. To improve reliability, we measured glucose with both CGM and SMBG at each visit and rescheduled tests if pre-meal values differed by more than 10 mg/dL. Nevertheless, repeated testing is necessary in future studies to more accurately assess individual variability.

Third, we focused on glucose dip as a measure of postprandial decline but did not assess time to minimum glucose. While glucose dip reflects the magnitude of glycemic decline-particularly relevant for liquid meals- time to minimum offers a different dimension of response. Future studies should consider both measures to more fully capture postprandial dynamics.

Lastly, while individual variability in response to ASBs was observed, the underlying mechanisms-including gut microbiota composition, insulin sensitivity, and neurological factors-were not directly assessed. Additionally, the specific type and composition of artificial sweeteners were not analyzed. Zero cola is primarily sweetened with sucralose and acesulfame potassium, and since different artificial sweeteners have been linked to varying physiological effects, their metabolism may contribute to individual differences in glycemic responses. Future studies should further explore these factors to better understand why certain individuals exhibit unexpected glycemic responses to ASBs.

## Conclusions

This study provides evidence that SSBs induce significant postprandial glucose dips, whereas mixed meals mitigate this effect by stabilizing glycemic response, rather than simply reflecting the sum of individual food components. While ASBs did not elicit a significant glycemic response in most participants, individual variability suggests that ASBs may not be metabolically neutral for everyone. These findings highlight the need for an individualized, meal-based approach to dietary management, as neither single food items nor standardized dietary guidelines for SSB and ASB consumption account for these variations in glycemic response.

## Electronic supplementary material

Below is the link to the electronic supplementary material.

Supplementary Material 1

## Data Availability

No datasets were generated or analysed during the current study.

## References

[1] Malik VS, Hu FB. Sugar-sweetened beverages and cardiometabolic health: an update of the evidence. Nutrients. 2019;11(8):1840. 31398911 10.3390/nu11081840 PMC6723421

[2] Tseng T-S, et al. Sugar intake from sweetened beverages and diabetes: a narrative review. World J Diabetes. 2021;12(9):1530–8. 34630905 10.4239/wjd.v12.i9.1530 PMC8472506

[3] Shin S, et al. Sugar-sweetened beverage consumption in relation to obesity and metabolic syndrome among Korean adults: a cross-sectional study from the 2012–2016 Korean National Health and Nutrition Examination Survey (KNHANES). Nutrients. 2018;10(10):1467. 30304842 10.3390/nu10101467 PMC6213560

[4] Malik VS, et al. Long-term consumption of sugar-sweetened and artificially sweetened beverages and risk of mortality in US adults. Circulation. 2019;139(18):2113–25. 30882235 10.1161/CIRCULATIONAHA.118.037401 PMC6488380

[5] Yang B, et al. Added sugar, sugar-sweetened beverages, and artificially sweetened beverages and risk of cardiovascular disease: findings from the women’s health initiative and a network meta-analysis of prospective studies. Nutrients. 2022;14(20):4226. 36296910 10.3390/nu14204226 PMC9609206

[6] Mullee A, et al. Association between soft drink consumption and mortality in 10 European countries. JAMA Intern Med. 2019;179(11):1479. 31479109 10.1001/jamainternmed.2019.2478 PMC6724165

[7] Yin J, et al. Intake of sugar-sweetened and low-calorie sweetened beverages and risk of cardiovascular disease: a meta-analysis and systematic review. Adv Nutr. 2021;12(1):89–101. 32696948 10.1093/advances/nmaa084 PMC7850046

[8] Qin P, et al. Sugar and artificially sweetened beverages and risk of obesity, type 2 diabetes mellitus, hypertension, and all-cause mortality: a dose–response meta-analysis of prospective cohort studies. Eur J Epidemiol. 2020;35(7):655–71. 32529512 10.1007/s10654-020-00655-y

[9] Tey SL, et al. Effects of aspartame-, monk fruit-, stevia- and sucrose-sweetened beverages on postprandial glucose, insulin and energy intake. Int J Obes (Lond). 2017;41(3):450–7. 27956737 10.1038/ijo.2016.225

[10] Kim Y, Keogh JB, Clifton PM. Consumption of a beverage containing aspartame and acesulfame K for two weeks does not adversely influence glucose metabolism in adult males and females: a randomized crossover study. Int J Environ Res Public Health. 2020;17(23):9049. 33291649 10.3390/ijerph17239049 PMC7731387

[11] Solomi L, Rees GA, Redfern KM. The acute effects of the non-nutritive sweeteners aspartame and acesulfame-K in UK diet cola on glycaemic response. Int J Food Sci Nutr. 2019;70(7):894–900. 30892106 10.1080/09637486.2019.1585418

[12] Bonnet F, et al. Consumption of a carbonated beverage with high-intensity sweeteners has no effect on insulin sensitivity and secretion in nondiabetic adults. J Nutr. 2018;148(8):1293–9. 29982723 10.1093/jn/nxy100

[13] McGlynn ND, et al. Association of low- and no-calorie sweetened beverages as a replacement for sugar-sweetened beverages with body weight and cardiometabolic risk. JAMA Netw Open. 2022;5(3):e222092. 35285920 10.1001/jamanetworkopen.2022.2092 PMC9907347

[14] Tobiassen PA, Køster-Rasmussen R. Substitution of sugar-sweetened beverages with non-caloric alternatives and weight change: a systematic review of randomized trials and meta-analysis. Obes Rev. 2024;25(2):e13652. 37880814 10.1111/obr.13652

[15] Dalenberg JR, et al. Short-term consumption of sucralose with, but not without, carbohydrate impairs neural and metabolic sensitivity to sugar in humans. Cell Metabol. 2020;31(3):493–e5027. 10.1016/j.cmet.2020.01.014 PMC7784207 32130881

[16] Yunker AG, et al. Obesity and sex-related associations with differential effects of sucralose vs sucrose on appetite and reward processing. JAMA Netw Open. 2021;4(9):e2126313. 34581796 10.1001/jamanetworkopen.2021.26313 PMC8479585

[17] Ebbeling CB et al. Effects of sugar-sweetened, artificially sweetened, and unsweetened beverages on cardiometabolic risk factors, body composition, and sweet taste preference: a randomized controlled trial. J Am Heart Association. 2020;9(15):e015668. 10.1161/JAHA.119.015668 PMC7792240 32696704

[18] Zeevi D, et al. Personalized nutrition by prediction of glycemic responses. Cell. 2015;163(5):1079–94. 26590418 10.1016/j.cell.2015.11.001

[19] Berry SE, et al. Human postprandial responses to food and potential for precision nutrition. Nat Med. 2020;26(6):964–73. 32528151 10.1038/s41591-020-0934-0 PMC8265154

[20] Danne T, et al. International consensus on use of continuous glucose monitoring. Diabetes Care. 2017;40(12):1631–40. 29162583 10.2337/dc17-1600 PMC6467165

[21] Brouns F, et al. Glycaemic index methodology. Nutr Res Rev. 2005;18(1):145–71. 19079901 10.1079/NRR2005100

[22] Wyatt P, et al. Postprandial glycaemic dips predict appetite and energy intake in healthy individuals. Nat Metabolism. 2021;3(4):523–9. 10.1038/s42255-021-00383-x PMC7610681 33846643

[23] Kim J, et al. A free-living setting, obesity is associated with greater food intake in response to a similar premeal glucose nadir. J Clin Endocrinol Metab. 2019;104(9):3911–9. 31087061 10.1210/jc.2019-00240 PMC6667277

[24] Pittas AG, et al. Interstitial glucose level is a significant predictor of energy intake in free-living women with healthy body weight. J Nutr. 2005;135(5):1070–4. 15867283 10.1093/jn/135.5.1070

[25] Wang J, et al. Consumption of added sugars from liquid but not solid sources predicts impaired glucose homeostasis and insulin resistance among youth at risk of obesity. J Nutr. 2014;144(1):81–6. 24198307 10.3945/jn.113.182519

[26] Drewnowski A, Bellisle F. Liquid calories, sugar, and body weight. Am J Clin Nutr. 2007;85(3):651–61. 17344485 10.1093/ajcn/85.3.651

[27] Horowitz M, et al. Gastric emptying in diabetes: clinical significance and treatment. Diabet Med. 2002;19(3):177–94. 11918620 10.1046/j.1464-5491.2002.00658.x

[28] Flint A, et al. Associations between postprandial insulin and blood glucose responses, appetite sensations and energy intake in normal weight and overweight individuals: a meta-analysis of test meal studies. Br J Nutr. 2007;98(1):17–25. 17524176 10.1017/S000711450768297X

[29] Guizar-Heredia R, et al. A new approach to personalized nutrition: postprandial glycemic response and its relationship to gut microbiota. Arch Med Res. 2023;54(3):176–88. 36990891 10.1016/j.arcmed.2023.02.007

[30] Fisher RS, et al. Gastric emptying of a physiologic mixed solid-liquid meal. Clin Nucl Med. 1982;7(5):215–21. 7083695 10.1097/00003072-198205000-00005

[31] Tey SL, et al. Effects of non-nutritive (artificial vs natural) sweeteners on 24-h glucose profiles. Eur J Clin Nutr. 2017;71(9):1129–32. 28378852 10.1038/ejcn.2017.37

[32] Gauthier E, Milagro FI, Navas-Carretero S. Effect of low-and non-calorie sweeteners on the gut microbiota: a review of clinical trials and cross-sectional studies. Nutrition. 2024;117:112237. 37897982 10.1016/j.nut.2023.112237

[33] Suez J, et al. Personalized microbiome-driven effects of non-nutritive sweeteners on human glucose tolerance. Cell. 2022;185(18):3307–e332819. 35987213 10.1016/j.cell.2022.07.016

[34] Kochem MC, Hanselman EC, Breslin PAS. Activation and inhibition of the sweet taste receptor TAS1R2-TAS1R3 differentially affect glucose tolerance in humans. PLoS ONE. 2024;19(5):e0298239. 38691547 10.1371/journal.pone.0298239 PMC11062524

[35] Shil A et al. Artificial sweeteners disrupt tight junctions and barrier function in the intestinal epithelium through activation of the sweet taste receptor, *T1R3*. Nutrients. 2020;12(6):1826. 10.3390/nu12061862 PMC7353258 32580504

[36] Kossiva L et al. Chronic use of artificial sweeteners: pros and cons. Nutrients. 2024;16(18):3162. 10.3390/nu16183162 PMC11435027 39339762

[37] Hutchins KM et al. Continuous glucose monitor overestimates glycemia, with the magnitude of bias varying by postprandial test and individual - a randomized crossover trial. Am J Clin Nutr. 2025;121(5):1025–34. 10.1016/j.ajcnut.2025.02.024 PMC12107490 40021059

[38] Hengist A, et al. Imprecision nutrition? Intraindividual variability of glucose responses to duplicate presented meals in adults without diabetes. Am J Clin Nutr. 2025;121(1):74–82. 39755436 10.1016/j.ajcnut.2024.10.007 PMC11747189

