# Supplementary material for: Glycemic response to SSBs and ASBs: the role of mixed meals and individual variability
Source: Nutr J. 2025 Jul 16;24:113. doi: 10.1186/s12937-025-01181-x (PMC12269098; doi:10.1186/s12937-025-01181-x)
Supplement: Supplementary file 1 — Supplementary Material 1 [file 12937_2025_1181_MOESM1_ESM.pdf]

Glycemic Response to SSBs and ASBs: The Role of Mixed Meals and Individual Variability

Sejin Kim, YoonJu Song

Supplementary Information

Supplementary Figure 1. Study design and timeline

Supplementary Figure 2. Flow chart of the 2-week intervention using CGM

Supplementary Figure 3. Clarke error grid analysis Comparing CGM and SMBG Measurements

Supplementary Table 1. Nutritional composition and test meals

Supplementary Table 2. Postprandial glycemic response by test meals

Supplementary Table 3. Meal order by test meals

Supplementary Table 4. Effects of meal type, meal order, and testing day on postprandial glycemic response from the linear mixed-effects model

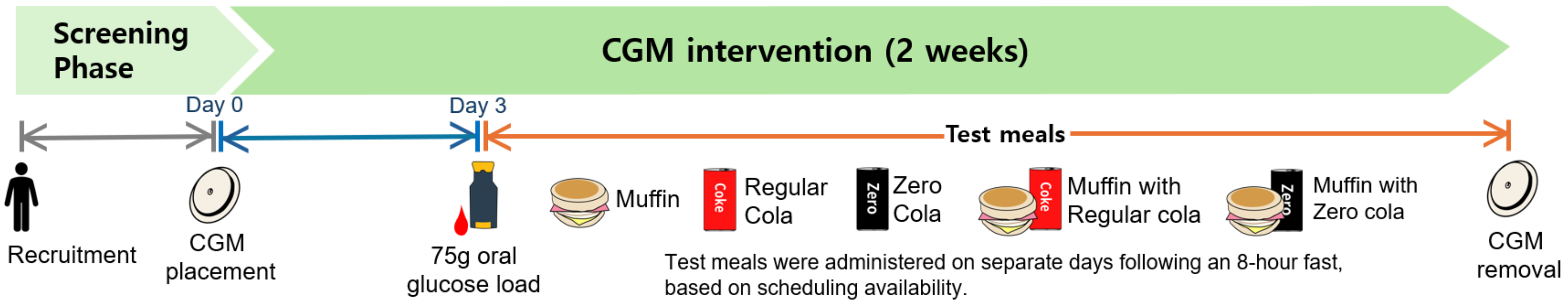

Supplementary Figure 1. Study design and timeline.

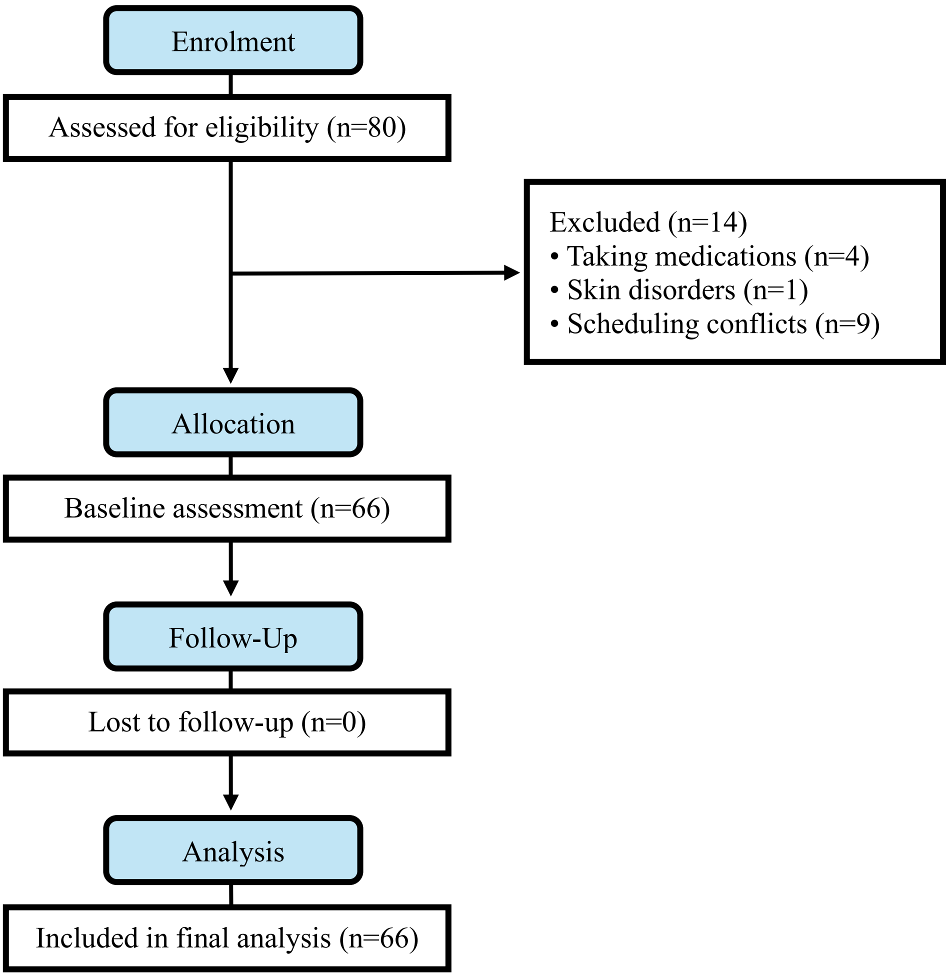

Supplementary Figure 2. Flow chart of the 2-week intervention using CGM

Supplementary Table 1. Nutritional composition of test meals

	75g oral glucose load (G)	Muffin (M)	Regular Cola (RC)	Zero Cola (ZC)	Muffin + RC (MRC)	Muffin + ZC (MZC)
Amount (g)	75.0	140.0	215.0	215.0	355.0	355.0
Energy (kcal)	300.0	283.0	92.0	0.0	375.0	283.0
Carbohydrate (g)	75.0	31.0	23.0	0.0	54.0	31.0
(Sugars, g)	(75.0)	(2.0)	(23.0)	(0.0)	(25.0)	(2.0)
(Fiber, g)		(3.8)			(3.8)	(3.8)
Protein (g)	0.0	15.0	0.0	0.0	15.0	15.0
Fat (g)	0.0	11.0	0.0	0.0	11.0	11.0
Energy from						
Carbohydrate (%)	100.0	43.8	100.0	0.0	57.6	43.8
Protein (%)	0.0	35.0	0.0	0.0	26.4	35.0
Fat (%)	0.0	21.2	0.0	0.0	16.0	21.2

Supplementary Table 2. Postprandial glycemic response by test meals

**PPGR (mg/dL)**	75g oral glucose load	Muffin	Regular cola	Zero cola	MRC	MZC
0 min	93.5 ± 9.6	89.4 ± 8.9	92.2 ± 9.8	90.1 ± 8.1	93.2 ± 8.8	95.4 ± 8.7
15 min	106.4 ± 13.3	94.1 ± 10.5	104.9 ± 13.9	89.3 ± 7.8	105.8 ± 14.4	102.0 ± 11.2
30 min	137.3 ± 20.8	110.7 ± 13.9	122.0 ± 17.8	88.8 ± 7.7	131.3 ± 21.0	119.2 ± 16.0
45 min	149.0 ± 25.9	119.8 ± 19.1	112.0 ± 18.6	89.7 ± 9.2	135.2 ± 28.2	127.0 ± 17.4
60 min	140.5 ± 29.7	112.0 ± 21.1	92.5 ± 17.5	90.0 ± 8.7	123.9 ± 26.7	119.6 ± 18.9
75 min	131.9 ± 29.5	103.1 ± 16.8	87.4 ± 16.1	89.7 ± 9.0	111.2 ± 20.3	110.0 ± 16.1
90 min	124.3 ± 25.1	97.3 ± 14.6	87.8 ± 11.7	89.4 ± 9.1	105.2 ± 17.8	104.0 ± 13.2
105 min	115.6 ± 20.8	94.8 ± 13.7	88.3 ± 11.4	88.6 ± 8.7	100.8 ± 15.9	100.1 ± 11.5
120 min	108.6 ± 18.8	93.8 ± 13.4	89.3 ± 11.3	88.5 ± 9.7	100.2 ± 14.6	99.0 ± 12.8
150 min	99.5 ± 20.8	91.9 ± 11.7	88.8 ± 9.5	88.7 ± 9.3	96.1 ± 13.1	96.0 ± 10.0
180 min	86.9 ± 18.1	88.9 ± 10.0	89.3 ± 8.3	88.4 ± 8.5	92.3 ± 13.3	94.3 ± 9.2
**Lowest glucose**	80.2 ± 13.2	82.9 ± 9.2	78.6 ± 9.3	84.0 ± 7.9	85.1 ± 10.6	89.5 ± 8.6
**Peak glucose**	155.7 ± 25.0	124.4 ± 18.3	128.9 ± 15.3	95.4 ± 10.1	145.1 ± 22.5	130.6 ± 16.6
**Incremental AUC (mmol/L)**						
120 min	216.4 ± 97.6	95.4 ± 49.9	65.0 ± 37.9	10.5 ± 26.7	142.4 ± 76.9	101.0 ± 52.7
180 min	251.2 ± 120.1	111.1 ± 59.5	72.8 ± 50.4	17.6 ± 43.7	161.6 ± 95.3	113.3 ± 58.4
**Total AUC (mmol/L)**						
120 min	837.6 ± 117.4	685.5 ± 78.3	654.1 ± 63.2	595.1 ± 51.5	757.6 ± 92.8	731.7 ± 75.5
180 min	1,166.5 ± 155.2	990.3 ± 101.8	950.3 ± 82.7	890.0 ± 78.0	1,078.1 ± 124.5	1,052.5 ± 97.5
**Glucose dip (%)**						
120 min	-2.4 ± 5.1	-4.6 ± 6.6	-13.7 ± 8.8	-5.5 ± 5.1	-5.1 ± 9.2	-3.8 ± 6.1
180 min	-14.0 ± 12.3	-7.1 ± 6.8	-14.5 ± 8.4	-6.6 ± 5.5	-8.4 ± 9.4	-6.0 ± 6.2

PPGR, postprandial glycemic response; MRC, muffin with regular cola; MZC, muffin with zero cola; AUC, area under the curve.

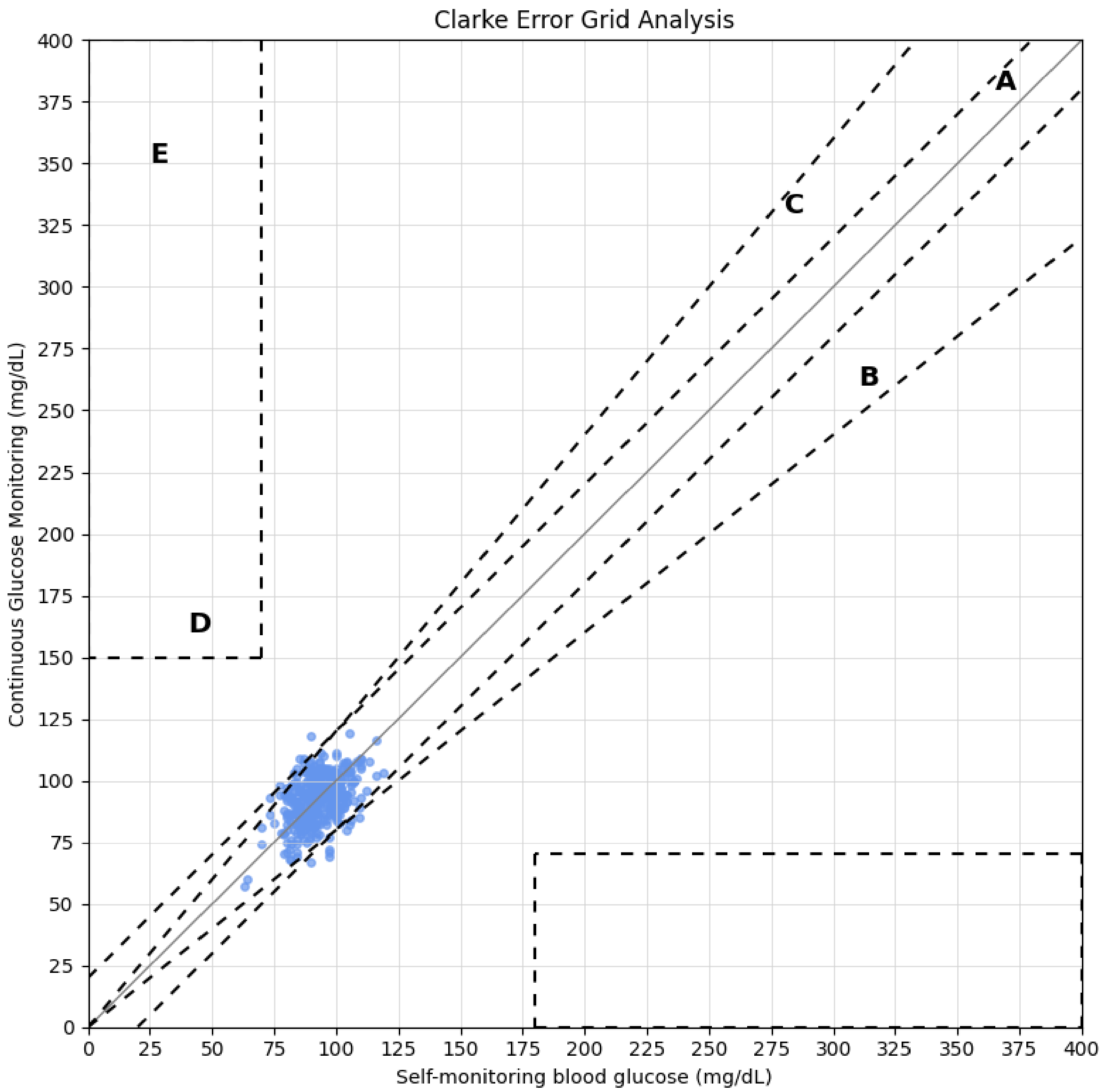

Supplementary Figure 3. Clarke error grid analysis comparing CGM and SMBG measurements

Clarke error grid analysis based on a total of 396 paired SMBG-CGM measurements showed that the CGM system demonstrated high clinical accuracy and reliability compared to self-monitored blood glucose (SMBG). Among all measurements, 96.2% fell within zone A, and 96.7% were within the clinically acceptable zones A and B, while only 3.3% were distributed across zones C through E.

Supplementary Table 3. Meal order by test meals

ID	G	M	RC	ZC	MRC	MZC	ID	G	M	RC	ZC	MRC	MZC
AS301	6	2	3	1	4	5	AS334	1	2	3	4	5	6
AS302	5	2	4	1	3	6	AS335	1	2	3	4	5	6
AS303	5	2	4	1	3	6	AS336	1	2	3	4	5	6
AS304	6	2	3	1	4	5	AS337	6	1	2	3	4	5
AS305	6	2	3	1	4	5	AS338	1	2	3	4	5	6
AS306	6	2	3	1	4	5	AS339	1	2	3	4	5	6
AS307	5	2	3	1	4	6	AS340	6	1	2	3	4	5
AS308	5	3	2	1	4	6	AS341	1	2	3	4	5	6
AS309	6	2	3	1	4	5	AS342	1	2	3	4	5	6
AS310	6	2	3	1	5	4	AS343	1	2	3	4	5	6
AS311	1	2	3	4	5	6	AS344	1	2	3	4	5	6
AS312	1	2	3	4	5	6	AS345	1	2	3	4	5	6
AS313	1	2	3	4	5	6	AS346	1	2	3	4	5	6
AS314	1	2	6	3	4	5	AS347	1	2	3	4	5	6
AS315	6	2	1	3	4	5	AS348	1	2	3	4	5	6
AS316	6	1	2	3	4	5	AS349	1	2	3	4	5	6
AS317	1	2	3	5	6	4	AS350	1	2	3	4	5	6
AS318	1	2	3	4	5	6	AS351	6	1	2	3	4	5
AS319	1	2	3	4	5	6	AS352	1	2	3	4	5	6
AS320	5	6	1	2	3	4	AS353	1	2	3	4	5	6
AS321	1	2	3	4	5	6	AS354	1	2	3	4	5	6
AS322	1	2	3	4	5	6	AS355	1	2	3	4	5	6
AS323	1	2	3	4	5	6	AS356	6	1	2	3	4	5
AS324	1	2	3	4	5	6	AS357	1	2	3	4	5	6
AS325	6	1	2	3	4	5	AS358	1	2	3	4	5	6
AS326	1	2	3	4	5	6	AS359	6	1	2	3	4	5
AS327	1	2	3	4	5	6	AS360	1	2	3	4	5	6
AS328	1	2	3	4	5	6	AS361	1	2	3	4	5	6
AS329	1	2	3	4	5	6	AS362	1	2	3	4	5	6
AS330	1	2	3	4	5	6	AS363	1	2	3	4	5	6
AS331	1	2	3	4	5	6	AS364	1	2	3	4	5	6
AS332	6	1	2	3	4	5	AS365	6	1	2	3	4	5
AS333	1	2	3	4	5	6	AS366	1	2	3	4	5	6

G, 75g oral glucose load; M, muffin; RC, regular cola, ZC, zero cola; MRC, muffin with regular cola; MZC, muffin with zero cola. Meal order indicates the sequence (1 to 6) in which the six test meals were consumed.

Supplementary Table 4. Effects of meal type, meal order, and testing day on postprandial glycemic response from the linear mixed-effects model

Effect	Num DF	Den DF	F value	p value
meal_type	5	276	12.90	<.0001
meal_order	5	276	1.87	0.1001
Time (testing day)	11	276	0.73	0.7091
meal_type*meal_order	8	276	1.17	0.3158
meal_type*time	20	276	0.84	0.6607

The model included a random intercept for participants and used a compound symmetry covariance structure. The likelihood ratio test comparing the full model to a null model without random effects was statistically significant ($p < 0.0001$), supporting the inclusion of subject-level variability.
